# Development of a WebPortal to Advance and Mobilize Knowledge Relevant to Trans-Affirming Care for Sexual Assault Survivors in Ontario, Canada

**DOI:** 10.1089/trgh.2020.0184

**Published:** 2022-08-01

**Authors:** Janice Du Mont, Sarah Daisy Kosa, Joseph Friedman Burley, Sheila Macdonald

**Affiliations:** ^1^ Women's College Research Institute, Women's College Hospital, Toronto, Canada.; ^2^ Social and Behavioural Health Sciences, Dalla Lana School of Public Health, University of Toronto, Toronto, Canada.; ^3^ Ontario Network of Sexual Assault/Domestic Violence Treatment Centres, Toronto, Canada.

**Keywords:** intersectoral, knowledge mobilization, network, online, sexual assault, transgender

## Abstract

Transgender persons experience high rates of sexual assault and often encounter providers who lack the knowledge to deliver appropriate postassault care and supports. To build capacity within health care and social service organizations supporting trans survivors of sexual assault across Ontario, Canada, we undertook a study to inform the development of a WebPortal intended to provide freely accessible resources relevant to the provision of trans-affirming care. In this survey, 70 representatives from community and health care organizations indicated their overall dissatisfaction with the information currently available on the care and support of trans survivors and identified a need for improved access to a range of resources.

## Introduction

Transgender (trans) persons face major health care disparities, with studies across disciplines demonstrating that barriers to care for this group frequently stem from a lack of knowledge among providers and noninclusive, discriminatory health care environments.^1–4^

Indeed, the 2019 Canadian Trans PULSE survey of 2873 trans (including nonbinary) people 14 years of age and older found that 45% of respondents had unmet health care needs in the past year, compared to 4% of the general population.^5^ Similar challenges to accessing appropriate supports have been documented across social service settings, including, but not limited to, those focused on housing, mental health, substance use, and social security.^4,6^ Whether within health care or social services, these obstacles are often exacerbated for trans survivors of sexual violence, who may experience complex postassault needs related to differing body configurations, histories of depression, and a lack of social supports, among other factors.^7^ In Canada, trans persons are significantly impacted by sexual assault, with 26% having reported an experience of victimization in the past 5 years.^8^

To improve care and supports for trans survivors of sexual assault in Ontario, Canada, a multiphase program of research was undertaken to build a province-wide intersectoral network of trans-positive health care services, including hospital-based sexual assault/domestic violence treatment centers, and community organizations (entitled the trans-LINK Network). In the initial planning stages of the Network, 106 representatives of these organizations provided key insights on priority areas for Network development and focus, of which the most prominent were education and training, peer involvement, advocacy, accessibility, and knowledge sharing and exchange.^9^ Based on these insights, a survey was distributed to all representatives to determine their priorities for specific Network activities and deliverables and solidify their status as Network members.^10,11^ Survey results highlighted that, for a large majority of responding organizations (87.8%), the creation of a knowledge sharing portal to share organizational information, updates, research developments, and other resources was a priority.^10^ This was an important finding as, to our knowledge, no other resource hub focused on trans-affirming care for sexual assault survivors has been available to service providers in this and other Canadian provinces. Existing resources internationally have tended to focus either on supporting survivors of violence or supporting trans persons, but rarely both. It is precisely because trans survivors often experience intersecting forms of discrimination, complex postassault needs, and barriers to appropriate care and support that a WebPortal focused specifically on trans-affirming care in Ontario is necessary.

In this study, our objective was to engage representatives from current Network member organizations in the development of a WebPortal. Based on the findings, the WebPortal will be designed to build capacity within and across service and support organizations by expanding free access to trans-affirming resources that “recognize, account for, and address the unique experiences and needs of trans persons” who have experienced sexual assault.^12, p.2^ The engagement of Network members in the design of the WebPortal will be vital in promoting its relevance and utility and is consistent with recommendations in the literature for consulting users in the early development of digital interventions to promote health equity for marginalized groups.^13,14^ This approach could be applied in other jurisdictions aspiring to enhance access to appropriate, context-specific online resources for improving the care available to trans survivors and those working with them.

## Methods

This study was approved by the Women's College Hospital Research Ethics Board (REB#2019-0073-E) and supported by an Advisory Committee representing trans community members and allies with extensive expertise in trans violence prevention and health (see Acknowledgments section for Advisory Committee membership).

### Sample

Seventy Network members consented to complete an online survey to inform the content of the WebPortal.

### Survey

The survey content was guided by previous research in WebPortal design^15,16^ and insights from our Advisory Committee.

The survey collected sociodemographic characteristics, including gender identity (woman, man, bigender, trans man, trans woman, transfeminine, transmasculine, genderqueer, agender, nonbinary, gender fluid, Two Spirit, and other), sexual orientation (lesbian, gay, bisexual, queer, Two Spirit, pansexual, asexual, heterosexual, and other), age group in years (19–24, 25–34, 35–44, 45–59, 60+), and ethnicity/racial background mostly identify with (Arab, West Asian, Black, Chinese, Filipino, Indigenous [e.g., Inuit, Métis, First Nations], Japanese, Korean, Latin American, South Asian [e.g., East Indian, Pakistani, Sri Lankan], and Southeast Asian, White, other). The survey also collected the characteristics of respondents' organizations, including the services and supports provided (e.g., counseling/mental health) and location by region within Ontario.

The survey captured access to and evaluation of online information on the care/support of trans survivors. Access was assessed on a 5-point Likert scale in response to the statement, “I am able to efficiently find information on care/support of trans survivors of sexual assault on the Internet” (1—strongly disagree, 2—disagree, 3—neither disagree nor agree, 4—agree, and 5—strongly agree). Evaluation was assessed on a 5-point Likert scale in response to the statement, “I trust the information on care/support of trans survivors of sexual assault on the Internet” (1—strongly disagree, 2—disagree, 3—neither disagree nor agree, 4—agree, and 5—strongly agree) and the question, “What is your overall satisfaction with information on care/support of trans survivors of sexual assault on the Internet?” (1—very unsatisfied, 2—somewhat unsatisfied, 3—neither unsatisfied nor satisfied, 4—somewhat satisfied, and 5—very satisfied).

Finally, the survey captured the importance rated on a 5-point Likert Scale (1—very unimportant, 2—somewhat unimportant, 3—neither unimportant nor important, 4—somewhat important, and 5—very important) of potential resources to include in the WebPortal (e.g., information/fact sheets, links to websites). The survey also asked, “Are there any specific resources or topics you would recommend be included on the WebPortal.” The survey was hosted on the online survey platform, SurveyMonkey.

### Data analysis

Data were exported from SurveyMonkey to IBM SPSS Version 25. Respondent and organizational characteristics, ratings for accessibility, trustworthiness, and satisfaction of internet information on the care/support of trans survivors, and ratings for perceived importance of potential WebPortal resources were summarized using descriptive statistics (frequencies, proportions). The perceived importance of each WebPortal resource was also summarized using means and standard deviations. Responses to the open-ended question were collated.

## Results

### Respondent and organizational characteristics

More than a half of respondents were aged 25–34 years (27.1%) or 35–44 years (25.7%; see Table 1). The majority (84.3%) identified as women; 8.6% as nonbinary; 4.3% as trans men; 2.9% each as transmasculine, genderqueer, and gender fluid; and/or 1.4% each as men, bigender, agender, and asog/tibo/pakilumang. Most respondents identified as heterosexual (60.0%); some identified as queer (20.0%), pansexual (8.6%), lesbian (7.1%), and/or bisexual (7.1%). Three quarters of respondents were white (74.3%); 5.7% identified as Indigenous, and 4.3% each as South Asian and Filipino.

**Table 1.** Respondent Characteristics

Item	*n*	%
Age group (*N*=70)		
19–24 Years	1	1.4
25–34 Years	19	27.1
35–44 Years	18	25.7
45–59 Years	25	35.7
60+ Years	4	5.7
Prefer not to answer	3	4.3
Gender identity^a^ (*N*=70)		
Woman	59	84.3
Man	1	1.4
Bigender	1	1.4
Trans man	3	4.3
Transmasculine	2	2.9
Genderqueer	2	2.9
Agender	1	1.4
Nonbinary	6	8.6
Gender fluid	2	2.9
Other (please specify): asog/tibo/pakilumang	1	1.4
Prefer not to answer	1	1.4
Sexual orientation^a^ (*N*=70)		
Lesbian	5	7.1
Gay	1	1.4
Bisexual	5	7.1
Queer	14	20.0
Two Spirit	1	1.4
Pansexual	6	8.6
Asexual	1	1.4
Heterosexual	42	60.0
Other (please specify): demisexual, panromantic	1	1.4
Ethnicity/racial background mostly identify with (*N*=70)		
Arab	1	1.4
Black	1	1.4
Chinese	1	1.4
Filipino	3	4.3
Indigenous	4	5.7
Korean	1	1.4
Latin American	1	1.4
South Asian	3	4.3
White	52	74.3
Prefer not to answer	3	4.3

^a^ Categories not mutually exclusive.

Respondents represented health care and community organizations providing a diverse range of supports, including lesbian, gay, bisexual, transgender, queer, Two Spirit+ (LGBTQ2S+) specific (22.1%), sexual assault specific (22.1%), counseling/mental health (19.1%), general health care (17.6%), advocacy and outreach (17.6%), violence (e.g., intimate partner violence; 16.2%), youth (10.3%), education and training (10.3%), housing/shelter (7.4%), HIV/AIDS (5.9%), immigration and settlement (2.9%), Indigenous (2.9%), legal (2.9%), employment (1.5%), and postsecondary student (1.5%). Organizations were located across all seven regions of Ontario: Central (23.5%), Central East (10.3%), Central West (14.7%), East (16.2%), Southwest (17.6%), Northeast (8.8%), and Northwest (8.8%).

### Accessibility and evaluation of internet information

Few respondents strongly agreed (1.5%) or agreed (13.6%) that they could efficiently find information on care/support of trans survivors of sexual assault on the internet (Table 2). No respondents strongly agreed and only 16.9% agreed that they trust online information on care/support of trans survivors. No respondents were very satisfied and just 10.8% were somewhat satisfied with the information on care/support of trans survivors available online.

**Table 2.** Accessibility and Evaluation of Internet Information

Item	Likert scale rating	*n*	%
Efficiently able to find information on care/support of trans survivors of sexual assault on the internet (*N*=66)	Strongly agree	1	1.5
	Agree	9	13.6
	Neither agree nor disagree	29	43.9
	Disagree	23	34.8
	Strongly disagree	4	6.1
Trust the information on care/support of trans survivors of sexual assault on the internet (*N*=65)	Strongly agree	0	0.0
	Agree	11	16.9
	Neither agree nor disagree	43	66.2
	Disagree	10	15.4
	Strongly disagree	1	1.5
Overall satisfaction with information on care/support of trans survivors of sexual assault on the internet (*N*=65)	Very satisfied	0	0.0
	Somewhat satisfied	7	10.8
	Neither satisfied nor unsatisfied	23	35.4
	Somewhat unsatisfied	24	36.9
	Very unsatisfied	11	16.9

### Prioritization of potential WebPortal resources

Except for “research opportunities/resources,” all potential resources on the survey received a mean rating of 4 or greater, indicating their importance for inclusion on the WebPortal (Table 3). These were information/fact sheets (mean=4.6), links to websites (4.5), guidelines (4.5), curricula/trainings (4.5), list of upcoming events (4.3), updates on ongoing Network activities (4.2), 1-to-2-page summaries of topics (4.2), policy briefs (4.2), archived webinars (4.1), membership directory (4.1), live webinars (4.1), and short videos/podcasts (4.0).

**Table 3.** Prioritization of Potential WebPortal Resources

Item	Likert scale rating	*n*	%	Mean	SD
Information/fact sheets (*N*=64)	Very important	48	75.0	4.6	0.9
	Somewhat important	12	18.8		
	Neither important nor unimportant	1	1.6		
	Somewhat unimportant	0	0.0		
	Very unimportant	3	4.7		
Links to websites (*N*=63)	Very important	40	63.5	4.5	0.9
	Somewhat important	20	31.7		
	Neither important nor unimportant	0	0.0		
	Somewhat unimportant	1	1.6		
	Very unimportant	2	3.2		
Guidelines (*N*=64)	Very important	42	65.6	4.5	0.9
	Somewhat important	15	23.4		
	Neither important nor unimportant	4	6.3		
	Somewhat unimportant	2	3.1		
	Very unimportant	1	1.6		
Curricula/trainings (e.g., instructions, materials; *N*=62)	Very important	42	67.7	4.5	1.0
	Somewhat important	13	21.0		
	Neither important nor unimportant	4	6.5		
	Somewhat unimportant	0	0.0		
	Very unimportant	3	4.8		
List of upcoming events (e.g., conferences, in-person trainings, webinars; *N*=63)	Very important	31	49.2	4.3	0.8
	Somewhat important	27	42.9		
	Neither important nor unimportant	3	4.8		
	Somewhat unimportant	0	0.0		
	Very unimportant	2	3.2		
Updates on ongoing Network activities (*N*=64)	Very important	26	40.6	4.2	0.9
	Somewhat important	31	48.4		
	Neither important nor unimportant	4	6.3		
	Somewhat unimportant	1	1.6		
	Very unimportant	2	3.1		
1-to-2 Page summaries of topics (*N*=63)	Very important	29	46.0	4.2	1.0
	Somewhat important	24	38.1		
	Neither important nor unimportant	5	7.9		
	Somewhat unimportant	2	3.2		
	Very unimportant	3	4.8		
Policy briefs (e.g., for advocacy, for use with policymakers; *N*=61)	Very important	24	39.3	4.2	0.8
	Somewhat important	25	41.0		
	Neither important nor unimportant	10	16.4		
	Somewhat unimportant	2	3.3		
	Very unimportant	0	0.0		
Archived webinars (*N*=64)	Very important	29	45.3	4.1	1.1
	Somewhat important	22	34.4		
	Neither important nor unimportant	6	9.4		
	Somewhat unimportant	5	7.8		
	Very unimportant	2	3.1		
Membership directory (*N*=63)	Very important	19	30.2	4.1	0.8
	Somewhat important	32	50.8		
	Neither important nor unimportant	10	15.9		
	Somewhat unimportant	2	3.2		
	Very unimportant	0	0.0		
Live webinars (*N*=64)	Very important	24	37.5	4.1	1.0
	Somewhat important	28	43.8		
	Neither important nor unimportant	6	9.4		
	Somewhat unimportant	4	6.3		
	Very unimportant	2	3.1		
Short videos/podcasts (*N*=62)	Very important	20	32.3	4.0	0.9
	Somewhat important	27	43.5		
	Neither important nor unimportant	10	16.1		
	Somewhat unimportant	4	6.5		
	Very unimportant	1	1.6		
Research opportunities/resources (e.g., grant, awards, potential partnerships; *N*=63)	Very important	19	30.2	3.9	1.0
	Somewhat important	27	42.9		
	Neither important nor unimportant	10	15.9		
	Somewhat unimportant	7	11.1		
	Very unimportant	0	0.0		

SD, standard deviation.

### Recommendations for specific content

The most common recommendation made by respondents for WebPortal content was a “directory of trans-friendly … professionals,” services, and supports in the community and health care sectors, including contact information and a description of in-person and virtual supports by region, which could be “provided to trans clients … especially [in] northern communities that may not have any trans specific resources.” Respondents noted that the directory could include listings for specific types of support, including “homelessness and housing resources safe for trans folks” and “legal supports.”

Some respondents suggested the inclusion of content on the WebPortal that would aid providers in better understanding the structural and systemic barriers to care faced by trans persons:

[H]ow sexual violence and gender-based discrimination … is rooted in colonialism and imperialism

[T]he (continued) history of medicine/social services pathologizing trans identities

[R]ecognizing and acknowledging cis privilege and cis-heteronormativity

[C]ritical reflexivity to support the provider [in] unpack[ing] their own cis-normative biases

A few emphasized the importance of “research and advocacy and information specific to trans BIPOC [Black, Indigenous, People of Color] survivors, communities, and services,” as well as “Two Spirit content” and material related to the “pediatric/adolescent population.”

A handful of respondents suggested that the WebPortal could include materials relevant to both service providers and trans survivors. These suggestions encompassed the areas of “sexual health” generally and access to specific supports, including having appropriate information on “crisis resources,” “medical care,” “counseling,” and “follow-up needs.” In addition, several respondents commented on the utility of including information for survivors on “navigating shelter/support programs and access issues,” “what to expect/how to advocate for [their] needs and comfort during [sexual assault] exam procedures,” “what [to] do if their rights are being violated by a service provider or organization,” and “experiences shared by trans folks.”

Other key suggestions for content included “guides on language … specific to both trans identity/experience AND gender based violence (using trauma-informed approach) for service providers,” resources on the provision of postsexual assault care, including “forensic assessment specific to gender [related] surgery,” and the “unique needs of trans survivors of DV [domestic violence] (chosen families, small communities of support that may overlap with the abuser, housing/food security, ability to access other support services, discrimination, etc.).” Also recommended was the inclusion of a “dictionary of terms,” “new journal articles,” and a listing of “socio-political movements and organizations to join or know about.”

## Summary and Conclusion

As identified by our representatives from diverse health care and social service organizations across Ontario, there is a clear need for streamlined access to reliable, high-quality online resources relevant to the care and support of trans survivors of sexual assault. These findings are consistent with previous research with queer and trans communities which highlighted a dearth in online resources and criticized existing resources for their poorer quality.^17–19^

In developing the WebPortal, it will be critical to integrate the array of resources rated as important by representatives as well as those suggested by representatives to improve the response to trans survivors from varied social locations (e.g., BIPOC, Two Spirit, and pediatric/adolescent populations). Furthermore, as identified by representatives in this study, resources included in the WebPortal should help raise critical awareness around, and thereby begin addressing, the structural and systemic barriers that often impinge on trans survivors' ability to access appropriate care and support (e.g., institutional transphobia). These findings are important as earlier studies have documented provider discrimination and a lack of knowledge related to diverse trans survivors' needs and experiences when navigating services.^20–23^

Several representatives also suggested that not all resources should be geared strictly toward providers (e.g., materials on crisis resources). This suggestion is helpful, as studies have indicated that repositories of online information can be a vital resource for trans persons, particularly youth.^17^ In addition, establishing a membership directory of trans-affirming supports and services may improve referrals within and across regions^11,24^ and could also facilitate direct access to safe, appropriate, and sensitive care for trans survivors. This could be particularly helpful where resources tend to be more scarce (e.g., in rural or northern communities).^25^

The insights garnered in this study will be critical in shaping the development of a WebPortal that is of practical utility to health care and social service professionals and the diverse trans survivors they support. Given the perceived lack of trustworthy online information related to the care/support of trans survivors, all resources included on the WebPortal will be screened for inclusion by experts in violence and trans health. In an iterative development process, knowledge users will be able to submit feedback on the WebPortal, which is intended to promote its ongoing relevance, appropriateness, and uptake. While this study was undertaken in the Ontario context, our user-driven approach to the creation and implementation of an online platform intended to support providers caring for trans survivors of sexual assault could be employed by others working to address complex public health issues with priority populations.

## Abbreviations Used

BIPOC: Black, Indigenous, People of Color
SD: standard deviation
